# Exploring the association between Frailty Index and Knee osteoarthritis in middle-aged and older Chinese adults: A cross-sectional analysis of data from the China Health and Retirement Longitudinal Study

**DOI:** 10.1371/journal.pone.0343370

**Published:** 2026-03-27

**Authors:** Na Zhu, Hongming Lin, Jia Yu, Rong Yang, Danhao Tao, Guoxin Huang, Da Qian, Yu Liu, Bingqian Chen

**Affiliations:** 1 Department of Rheumatology and Immunology, Changshu Hospital Affiliated to Soochow University, Changshu No.1 People’s Hospital, Changshu, China; 2 Department of Plastic Surgery, Xiangyang No.1 People’s Hospital, Hubei University of Medicine, Xiangyang, China; 3 Department of General Surgery, Changshu Hospital Affiliated to Soochow University, Changshu No.1 People’s Hospital, Changshu, China; 4 Department of Evidence-Based Medicine Center, Xiangyang No.1 People’s Hospital, Hubei University of Medicine, Xiangyang, China; 5 Department of Burn and Plastic Surgery-Hand Surgery, Changshu Hospital Affiliated to Soochow University, Changshu No.1 People’s Hospital, Changshu, China; 6 Department of Medical, Xiangyang No.1 People’s Hospital, Hubei University of Medicine, Xiangyang, Hubei, China; 7 Department of Orthopedics, Changshu Hospital Affiliated to Soochow University, Changshu No.1 People’s Hospital, Changshu, China; University College London, UNITED KINGDOM OF GREAT BRITAIN AND NORTHERN IRELAND

## Abstract

**Objective:**

To explore the relationship between Frailty Index (FI) and Knee osteoarthritis (KOA) in Chinese people aged 45 and above.

**Methods:**

Data on symptomatic KOA, FI, and over ten covariates were from the China Health and Retirement Longitudinal Study (CHARLS). Logistic regression and restricted cubic splines (RCS) were used to analyze the correlation. Subgroup and interaction analyses were also conducted, and FI was divided into quartiles to assess result stability. Six machine learning constructs were used to build predictive models, which were then evaluated.

**Results:**

Among 29,105 participants, 5,012 had symptomatic KOA. Weighted multivariate logistic results showed a correlation between KOA and frailty (OR 3.63(3.48,3.78)), present across quartiles. Subgroup analyses revealed potential effects of various factors on their association, with significant interactions found for gender, education, place of residence, alcohol consumption, hypertension, and dyslipidemia. RCS results indicated a logarithmic non-linear relationship between symptomatic KOA and frailty. The area under the curve of the Random Forest Machine model is 0.90, and it performs best on the calibration curve and decision curve analysis.

**Conclusion:**

This study showed a nonlinear relationship between FI and KOA in middle-aged and older Chinese adults, with higher FI scores linked to higher KOA prevalence and a significant inflection point, informing focused KOA interventions in aging populations.

## Introduction

Knee osteoarthritis (KOA) is a common joint disease due to knee cartilage damage, causing inflammation and pain, particularly in middle-aged and elderly people [1]. The multifactorial aetiology of KOA encompasses genetic susceptibility, lifestyle influences, gender, age, body mass index, and physical activity. Specifically, obesity, prolonged standing, and overuse of the knee joints have been demonstrated to accelerate the deterioration of articular cartilage, thereby increasing the risk of KOA [2]. More than 260 million people worldwide suffer from osteoarthritis of the knee, and the prevalence of the disease is expected to be as high as 15.7% by 2032 [3]. With the increasing aging of the population, the prevalence of KOA continues to rise, which has a serious impact on the quality of life of middle-aged and elderly people. At the same time, KOA not only causes great suffering to individual patients, but also imposes a heavy burden on the health-care system as well as on the socio-economy.

Frailty is a clinical syndrome closely linked to aging, characterized by dysfunctions in multiple physiological systems, including reduced muscle strength, endurance, balance, and cognitive abilities [4]. It significantly increases the risk of falls and fractures in older adults and is strongly associated with various adverse health outcomes such as hospitalization, functional decline, and mortality [5]. To quantitatively measure an individual’s frailty, the Frailty Index (FI) is widely utilized in geriatric research. The FI is calculated by summing the number of functional declines and disease-related losses in older adults, resulting in a continuous score that directly indicates the level of frailty [6].

In recent years, a significant amount of research has shown a strong correlation between Knee Osteoarthritis (KOA) and frailty [7,8]. Individuals with KOA often experience substantial decreases in muscle strength and function, mirroring the clinical signs of frailty. Moreover, the pain and mobility restrictions associated with KOA can further intensify weakness, leading to a negative feedback loop [7]. This correlation not only indicates a possible pathophysiological connection between KOA and frailty but also implies that these two conditions might share common pathogenic mechanisms or risk factors.

To fill the research gap in China, this study explores the relationship between Frailty Index (FI) and Knee osteoarthritis (KOA) in Chinese people aged 45 and above using data from the China Health and Retirement Longitudinal Study (CHARLS) [9].

## Materials and methods

### Data source

The data came from CHARLS, a large-scale survey by Peking University’s National Development Research Institute [9]. It collects data on Chinese people aged 45 and older, covering basic information, health status, living habits, and economic and social support. The studies involving humans were approved by Institutional Review Committee of Peking University. The studies were conducted in accordance with the local legislation and institutional requirements. The participants provided their written informed consent to participate in this study. This study was reported in strict accordance with the STROBE reporting guidelines and the TRIPOD+AI reporting guidelines.

### Acquisition of data on osteoarthritis of the knee

The source of data on osteoarthritis of the knee references the prior literature and was obtained primarily based on questionnaires [10]. In the 2011, 2013, and 2015 surveys, investigators asked participants, ‘Do you suffer from pain in any part of your body on a regular basis?’ If the participant answered in the affirmative, the next question was asked, ‘What part of your body do you feel pain in?’ when participants answered knee, they were diagnosed with osteoarthritis of the knee.

### Acquisition of frailty data

This study scientifically evaluated the Frailty Index (FI) of individuals, utilizing comprehensive data from the CHARLS database and adhering to a standardized research process. The FI assessment encompassed various dimensions such as basic and instrumental activities of daily living (e.g., personal hygiene, dressing, financial management), physical function limitations, chronic diseases, mental health indicators, and subjective health self-assessment. In constructing the FI, a binary coding system was employed, with ‘0’ signifying no deficit and ‘1’ indicating a deficit. For variables with intermediate states (e.g., ‘occasional’ or ‘possible’), a value of ‘0.5’ was assigned to denote a partial deficit. This coding method provides a more detailed and accurate representation of an individual’s health status [11]. Activities of daily living include Bathing, Dressing, Use of toilet, Transferring, Continence, Eating; Instrumental activities of daily living include Cooking, Shopping, Doing household, Taking medicine, Managing money; Physical functional limitations include Lift a weight of 5 kg, Walking 1 km, Walking 100m, Stooping, kneeling, or crouching, Able to stand up from sitting, Able to pick up a coin from a table, Running or jogging about 1 km, Reaching or extend arms, Climbing several flights of stairs without resting; Chronic disease include Chronic lung diseases (Chronic bronchitis, emphysema), Asthma, stroke, CVD, Gastric or duodenal ulcer, Kidney disease, Liver disease, Memory related disease (Dementia, brain atrophy, and Parkinson’s disease), Emotional, nervous, or psychiatric problems; Mental health include Feel depressed, Feel fearful, Feel happy, Feel everything was an effort, Feel could not get “going”; Subjective functioning include Self-rated health. For detailed content, please refer to S1 Appendix.

### Acquisition of covariates

When evaluating covariates, we considered socio-demographic traits, lifestyle habits, and chronic disease conditions. Socio-demographic traits comprised age, gender, residence type (rural or urban), education level (illiterate, junior high school or below, high school or above), and marital status (married, unmarried). Body mass index (BMI) was computed using WHO standards from height and weight measurements and categorized into underweight/normal, overweight, and obese. Regarding lifestyle, we assessed respondents’ smoking and alcohol consumption, categorizing them based on past use. For chronic diseases, we identified hypertension, diabetes, and dyslipidemia using self-reported medical histories and clinical tests. Hypertension was confirmed by self-reported diagnosis, medication, and blood pressure readings; diabetes by diagnosis, insulin or medication use, and blood glucose or HbA1c levels; and dyslipidemia by diagnosis, medication, and lipid profiles.

### Machine learning methods

The dataset was randomly partitioned into a training set (70%) and a validation set (30%). The training set was utilized for the initial construction and training of the model, while the validation set was reserved for the subsequent objective evaluation of model performance. Feature selection was performed employing a combination of the random forest algorithm and 5-fold cross-validation. Following each round of feature selection, the mean area under the receiver operating characteristic (ROC) curve (AUC) was calculated to identify the optimal subset of feature variables. The selected features were subsequently incorporated into model construction. Six representative machine learning algorithms were evaluated: Logistic Regression (LR), Support Vector Machine (SVM), Multilayer Perceptron (MLP), Light Gradient Boosting Machine (LightGBM), Extreme Gradient Boosting (XGBoost), and Random Forest (RF). For each algorithm, a grid search strategy was implemented to determine the optimal combination of hyperparameters, ensuring the model achieved its best predictive performance. Model performance was comprehensively assessed across multiple dimensions: discrimination, calibration, and clinical utility. The net clinical benefit across varying probability thresholds was evaluated using decision curve analysis (DCA). The six models were evaluated using a multi-dimensional assessment method, including accuracy, sensitivity, precision, specificity and F1.

### Statistical methods

In this study, participants’ basic characteristics were summarized using descriptive statistics, including means/medians and standard deviations/quartiles for continuous data, and percentages for categorical data. The Frailty Index (FI) was analyzed both continuously and divided into quartiles (Q1: ≤ 0.043, Q2: 0.043–0.081, Q3: 0.081–0.145, Q4: ≥ 0.145). Group differences were compared using t-tests for normal data, Wilcoxon tests for non-normal data, and chi-square tests for categorical data. The FI was scaled up by 10 in the continuity model. Logistic regression models estimated the association between FI and KOA, with Model 1 unadjusted, Model 2 adjusted for gender, age, and BMI, and Model 3 adjusted for all covariates. Restricted cubic spline regression explored the non-linear relationship between FI and KOA. The RCS analysis and inflection point identification in this study were performed using the rms package in R 4.1.2 software. In Model 3, which adjusted for all covariates, RCS models were constructed using the 5th and 95th percentiles of FI as inflection points. The inflection points of the frailty index were determined through likelihood ratio tests (P < 0.001) and validated by 1000 Bootstrap samples.

All analyses were conducted using R statistical software, version V.4.1.2 (http://www.R-project.org), and p-values less than 0.05 were considered statistically significant.

## Results

### Baseline information

**Fig 1:**
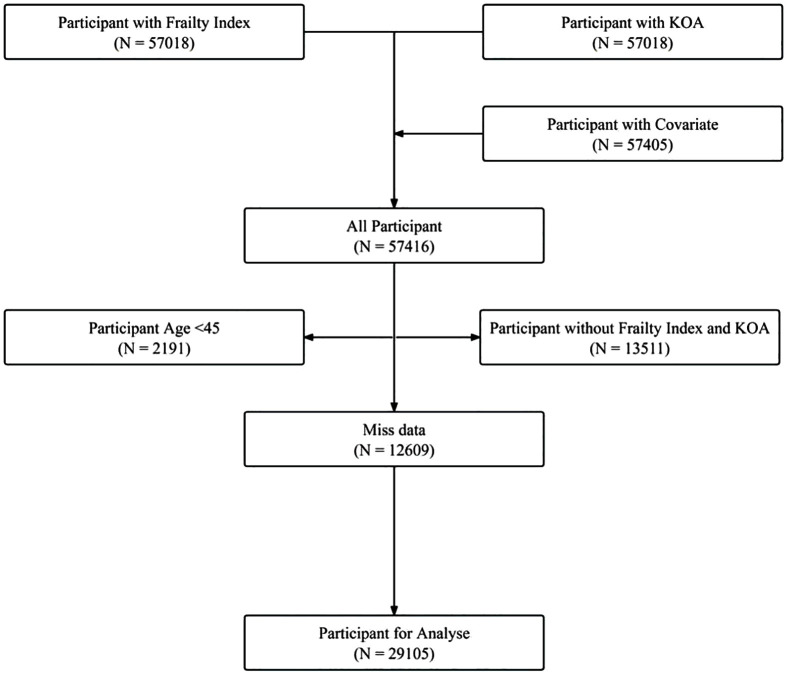
Screening flow chart.

Between 2011 and 2015, data on knee osteoarthritis was available for 57,018 participants, the same number had data on the frailty index, and 57,405 had data on relevant covariates, totaling 57,416 after data integration. Excluding those under 45 years left 55,225 participants. After removing those with missing covariate data, 29,105 participants remained, comprising 24,093 without knee osteoarthritis and 5,012 with the condition. The screening process is depicted in Fig 1. The non-KOA group included 12,274 (50.94%) females and 11,819 (49.06%) males, with an average age of 59.88 ± 9.54 years and a BMI of 23.81 ± 3.78. The KOA group had 3,437 (68.58%) females and 1,575 (31.42%) males, averaging 61.43 ± 9.13 years old with a BMI of 23.83 ± 4.02. Significant differences existed between the groups in age, sex, marital status, education level, residence, smoking, alcohol consumption, hypertension, diabetes, and dyslipidemia, as detailed in Table 1.

**Table 1:** Characteristics of participants.

Variable	Total (n = 29105)	No KOA (n = 24093)	KOA (n = 5012)	P value
Age,year,mean±SD	60.14 ± 9.49	59.88 ± 9.54	61.43 ± 9.13	<0.0001
BMI,mean±SD	23.81 ± 3.82	23.81 ± 3.78	23.83 ± 4.02	0.66
Sex,n (%)				<0.0001
Female	15711 (53.98)	12274 (50.94)	3437 (68.58)	
Male	13394 (46.02)	11819 (49.06)	1575 (31.42)	
Marital status,n (%)				<0.0001
Married	24055 (82.65)	20133 (83.56)	3922 (78.25)	
Non-Married	5050 (17.35)	3960 (16.44)	1090 (21.75)	
Education,n (%)				<0.0001
High school or above	3466 (11.91)	3168 (13.15)	298 (5.95)	
Illiterate	7688 (26.41)	5829 (24.19)	1859 (37.09)	
Junior high school	17951 (61.68)	15096 (62.66)	2855 (56.96)	
Residence,n (%)				<0.0001
Rural	17992 (61.82)	14399 (59.76)	3593 (71.69)	
Urban	11113 (38.18)	9694 (40.24)	1419 (28.31)	
Smoke,n (%)				<0.0001
No	21215 (72.89)	17250 (71.60)	3965 (79.11)	
Yes	7890 (27.11)	6843 (28.40)	1047 (20.89)	
Drinking,n (%)				<0.0001
No	19324 (66.39)	15614 (64.81)	3710 (74.02)	
Yes	9781 (33.61)	8479 (35.19)	1302 (25.98)	
Hypertension,n (%)				<0.0001
No	16738 (57.51)	14046 (58.30)	2692 (53.71)	
Yes	12367 (42.49)	10047 (41.70)	2320 (46.29)	
Diabetes,n (%)				<0.0001
No	25273 (86.83)	21082 (87.50)	4191 (83.62)	
Yes	3832 (13.17)	3011 (12.50)	821 (16.38)	
Dyslipidemia,n (%)				<0.0001
No	21127 (72.59)	17746 (73.66)	3381 (67.46)	
Yes	7978 (27.41)	6347 (26.34)	1631 (32.54)	
Frailty Index,mean±SD	0.11 ± 0.09	0.09 ± 0.07	0.20 ± 0.11	<0.0001

**BMI, Body Mass Index; FI, Frailty Index; KOA, Knee osteoarthritis.**

### Relationship between FI and KOA

As a continuous variable, the Frailty Index (FI) was identified as a risk factor for Knee Osteoarthritis (KOA) with an odds ratio (OR) of 3.54 (3.41, 3.68) in univariate logistic analysis. After adjusting for all covariates, the multivariate logistic analysis still indicated that FI is a risk factor for KOA, with an OR of 3.63 (3.48, 3.78), as detailed in Table 2.

**Table 2:** Relationship between FI and KOA.

Exposure	Model 1		Model2		Model 3	
	OR (95%CI)	P Value	OR (95%CI)	P Value	OR (95%CI)	P Value
Frailty Index	3.54 (3.41,3.68)	<0.0001	3.69 (3.54,3.84)	<0.0001	3.63 (3.48,3.78)	<0.0001
Quartile of Frailty Index						
Q1	Ref	Ref	Ref	Ref	Ref	Ref
Q2	3.06 (2.55, 3.67)	<0.0001	3.03 (2.53, 3.64)	<0.0001	2.96 (2.47, 3.56)	<0.0001
Q3	9.25 (7.85,10.90)	<0.0001	9.27 (7.86,10.94)	<0.0001	8.9 (7.54,10.50)	<0.0001
Q4	38.9 (33.19,45.59)	<0.0001	40.66 (34.59,47.81)	<0.0001	38.17 (32.43,44.92)	<0.0001
P for trend		<0.0001		<0.0001		<0.0001

When FI was categorized into quartiles, univariate logistic results showed a significantly higher OR of 38.9 (33.19, 45.59) for the fourth quartile (Q4) compared to the first quartile (Q1). After adjusting for all covariates, the multivariate logistic results revealed ORs of 2.96 (2.47, 3.56) for Q2, 8.9 (7.54, 10.50) for Q3, and 38.17 (32.43, 44.92) for Q4 relative to Q1. A linear trend between the Frailty Index and KOA was suggested (P for trend < 0.0001).

### Subgroup analyses

When the Frailty Index (FI) was considered as a continuous variable, differences were observed in gender, marital status, education, place of residence, smoking, alcohol consumption, hypertension, diabetes, and dyslipidemia, as shown in Table 3. These differences persisted when the FI was categorized into quartiles. Interaction analyses further revealed significant differences in gender (male vs. female), education level (high school or above, illiterate, junior high school), place of residence (rural vs. urban), alcohol consumption (no vs. yes), hypertension (no vs. yes), and dyslipidemia (no vs. yes).

**Table 3:** Subgroup analysis.

Character	Frailty Index			Quartile of Frailty Index						
	OR (95%CI)	P Value	P for Interaction	Q1	Q2		Q3		Q4	
					OR (95%CI)	P Value	OR (95%CI)	P Value	OR (95%CI)	P Value
Sex			0.01							
Male	3.23 (3.04,3.43)	<0.0001		Ref	3.94 (2.94, 5.35)	<0.0001	12.06 (9.26,15.99)	<0.0001	43.51 (33.68,57.29)	<0.0001
Female	3.59 (3.42,3.78)	<0.0001		Ref	2.43 (1.93, 3.08)	<0.0001	7.11 (5.81, 8.78)	<0.0001	31.35 (25.82,38.45)	<0.0001
Marital status			0.08							
Married	3.60 (3.44,3.76)	<0.0001		Ref	3.25 (2.68, 3.97)	<0.0001	9.41 (7.90,11.28)	<0.0001	39.16 (33.09,46.71)	<0.0001
Non-Married	3.30 (3.04,3.59)	<0.0001		Ref	2.08 (1.28, 3.47)	0.004	8.19 (5.44,12.91)	<0.0001	35.28 (23.82,54.91)	<0.0001
Education			<0.0001							
High school or above	3.69 (3.50,3.89)	<0.0001		Ref	3.16 (2.53, 3.99)	<0.0001	9.38 (7.67,11.58)	<0.0001	37.12 (30.55,45.57)	<0.0001
Illiterate	2.97 (2.80,3.16)	<0.0001		Ref	2.46 (1.70, 3.61)	<0.0001	7.11 (5.15,10.09)	<0.0001	31.40 (23.05,44.05)	<0.0001
Junior high school	4.95 (4.19,5.87)	<0.0001		Ref	3.08 (1.80, 5.39)	<0.0001	10.63 (6.69,17.67)	<0.0001	42.06 (26.71,69.54)	<0.0001
Residence			0.001							
Rural	3.20 (3.00,3.42)	<0.0001		Ref	2.94 (2.20, 3.98)	<0.0001	9.27 (7.16,12.19)	<0.0001	32.63 (25.45,42.53)	<0.0001
Urban	3.66 (3.49,3.84)	<0.0001		Ref	3.09 (2.45, 3.91)	<0.0001	9.00 (7.33,11.16)	<0.0001	40.33 (33.10,49.70)	<0.0001
Smoke			0.07							
No	3.45 (3.30,3.60)	<0.0001		Ref	2.77 (2.25, 3.43)	<0.0001	8.26 (6.88,10.01)	<0.0001	35.23 (29.53,42.41)	<0.0001
Yes	3.76 (3.46,4.09)	<0.0001		Ref	3.95 (2.74, 5.80)	<0.0001	12.30 (8.87,17.55)	<0.0001	47.90 (34.83,67.83)	<0.0001
Drinking			<0.0001							
No	3.26 (3.13,3.41)	<0.0001		Ref	2.72 (2.17, 3.44)	<0.0001	8.61 (7.04,10.63)	<0.0001	35.77 (29.48,43.89)	<0.0001
Yes	4.51 (4.15,4.90)	<0.0001		Ref	3.66 (2.73, 4.96)	<0.0001	10.10 (7.74,13.41)	<0.0001	43.06 (33.25,56.76)	<0.0001
Hypertension			<0.0001							
No	3.12 (2.96,3.29)	<0.0001		Ref	3.10 (2.27, 4.29)	<0.0001	10.06 (7.64,13.50)	<0.0001	41.02 (31.50,54.58)	<0.0001
Yes	4.15 (3.92,4.40)	<0.0001		Ref	3.06 (2.45, 3.84)	<0.0001	8.93 (7.32,10.98)	<0.0001	39.26 (32.41,48.02)	<0.0001
Diabetes			0.18							
No	3.34 (3.05,3.68)	<0.0001		Ref	5.56 (2.73, 12.86)	<0.0001	18.58 (9.69, 41.44)	<0.0001	79.03 (41.85,174.57)	<0.0001
Yes	3.59 (3.44,3.75)	<0.0001		Ref	2.94 (2.44, 3.56)	<0.0001	8.79 (7.44,10.45)	<0.0001	36.87 (31.40,43.60)	<0.0001
Dyslipidemia			<0.0001							
No	3.73 (3.56,3.92)	<0.0001		Ref	3.05 (2.46, 3.79)	<0.0001	9.44 (7.82,11.50)	<0.0001	39.63 (33.03,47.99)	<0.0001
Yes	3.14 (2.94,3.35)	<0.0001		Ref	3.05 (2.17, 4.36)	<0.0001	8.58 (6.33,11.89)	<0.0001	35.71 (26.68,49.01)	<0.0001

### Restricted cubic spline (RCS)

**Fig 2:**
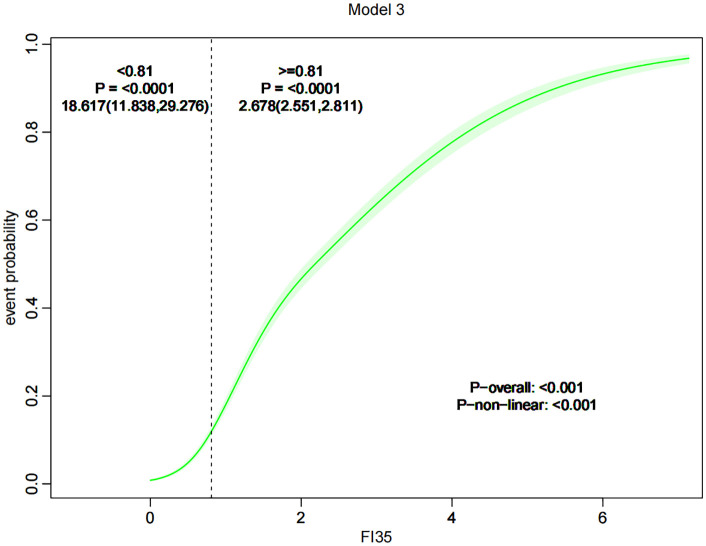
Trend graph of RCS changes.

RCS analysis was employed to investigate the potential non-linear relationship between the Frailty Index and Knee Osteoarthritis (KOA). The results indicated a significant non-linear relationship (P non-linear < 0.001), characterized by a logarithmic pattern. Using the median value of 0.81 as an inflection point, when the Frailty Index was below 0.81, the risk of developing KOA increased markedly with the Frailty Index (OR = 18.62(11.84, 29.28), P < 0.0001). However, when the Frailty Index exceeded 0.81, although the risk of developing KOA still rose with increasing Frailty Index, the rate of increase was reduced (OR = 2.68(2.55, 2.81), P < 0.0001), as illustrated in Fig 2.

### Machine learning results

**Fig 3:**
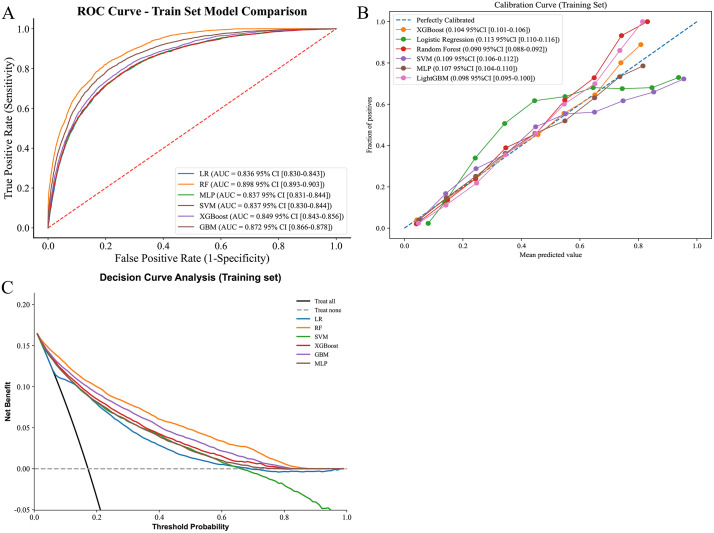
ROC curves and model evaluation of six ML models in traning set. **(A)**: ROC curves; **(B)**: Calibration Curves; **(C)**: Decision curves analysis.

This study divided the dataset into training and validation sets at a 7:3 ratio, with the training set comprising 20,373 subjects and the validation set comprising 8,732 subjects. Feature selection was performed using the Random Forest (RF) algorithm and cross-validation. Results indicated that incorporating four features—body mass index (BMI), frailty index, age, and education level—into the model improved its predictive performance as measured by the area under the curve (AUC) metric (detailed results are presented in S1 Fig.). Meanwhile, the baseline features for the training and validation sets have been compiled in Supplementary Table 1 to provide a reference for baseline consistency in subsequent analyses. This study further employed the aforementioned filtered features for constructing and conducting preliminary evaluations of multi-class machine learning models. Within the training set, the AUC values for the Random Forest (RF), LightGBM, and XGBoost models were relatively higher (Fig 3); In the validation set, LightGBM, XGBoost, and RF models also exhibited high AUC values (S2A Fig.). Calibration curve analysis revealed that the RF and LightGBM models demonstrated good fit between predictions and actual outcomes in the training set, while LightGBM, RF, and XGBoost models all showed superior fit in the validation set (Fig 3, S2B Fig.). Decision curve analysis (DCA) results indicate that across both training and validation sets, RF and LightGBM models demonstrate relatively stable performance and higher net benefit across a broad range of probability thresholds (Fig 3, S2C Fig.). The multi-dimensional evaluation results are presented in Table 4 and appendix Table 2.

**Table 4:** The multi-dimensional evaluation results for six different machine learning models in the training set.

Models	Accuracy	Sensitivity	Precision	Specificity	F1	AUC
SVM	0.85	0.35	0.62	0.96	0.45	0.84
XGBoost	0.86	0.35	0.65	0.96	0.46	0.85
LightGBM	0.86	0.38	0.69	0.96	0.49	0.87
Logistic Regression	0.84	0.16	0.67	0.98	0.26	0.84
Random Forest	0.88	0.42	0.75	0.97	0.54	0.90
MLP	0.85	0.35	0.61	0.95	0.45	0.84

## Discussion

Osteoarthritis (OA), a prevalent chronic condition in the elderly, frequently affects the knee joint. It is marked by persistent pain and reduced mobility, significantly impairing the quality of life. [12]. As China’s population ages, the incidence of OA is on the rise [13]. Concurrently, frailty syndrome, another age-related condition, is also leading to increased disability among the elderly. [14]. Both conditions share several common risk factors, including gender, obesity, place of residence, and education level. Our study identified a link between Frailty Index (FI) and the prevalence of Knee Osteoarthritis (KOA) in Chinese individuals aged 45 and above. Higher frailty correlated with a greater risk of developing KOA. Subgroup analyses also indicated that gender, marital status, education, place of residence, smoking, alcohol consumption, hypertension, diabetes, and dyslipidemia may influence the association between frailty and KOA. Interaction analyses highlighted significant associations involving gender (male vs. female), education (high school or above, illiterate, junior high school), residence (rural vs. urban), alcohol consumption (no vs. yes), hypertension (no vs. yes), and dyslipidemia (no vs. yes).

Prior research has delved into the intricate connection between Frailty Index (FI) and Knee Osteoarthritis (KOA). Notably, a significant knee OA cohort study, the Multicentre Osteoarthritis Study, found that the rise in frailty prevalence was most evident among patients with symptomatic or bilateral knee OA [15]. Similarly, in a European multicentre study of community-dwelling people aged 65–85 years from six countries, the risk of frailty in people with OA was between 1.5 and nearly three times higher [16]. The study found that frail people often have limited mobility in different environments and reduced muscle strength and mass [17]. These factors hinder their ability to perform basic daily tasks and participate in physical activities. The predominant clinical manifestation in people with osteoarthritis is pain, which plays an important role in health service use and clinical decision-making and is well articulated in biopsychosocial models [18]. This is because knee pain and quadriceps muscle weakness associated with knee OA can lead to reduced physical activity, which can result in loss of muscle mass (due to wasting) and weakness [19]. In turn, pain and instability themselves can lead to reduced physical activity, which can lead to a generalized loss of muscle mass and thus the development of frailty [15]. Shega JW et al. found that community-dwelling older people in the presence of knee pain had a 2-fold and 5-fold higher risk of frailty or weakness, respectively, compared with mild or no knee pain [20]. Kanapuru et al. reported that lower muscle mass may be a risk factor for knee pain in patients with mild knee OA on imaging, but not in patients with severe OA on imaging [21]. In females, high-fat mass and low lower extremity muscle mass were associated with the presence and severity of knee OA. Lower extremity muscle mass was more strongly associated with knee OA than obesity in women [22]. In a prospective study to predict pain, it was noted that approximately two-thirds of middle-aged and older adults are frail or weak, and that frailty may be an important therapeutic target for knee pain [23].

Another potential mechanism for the link between FI and KOA is through synergy between knee OA and systemic inflammation [24,25]. Studies showed higher circulating levels of pro-maternal cytokines such as IL6, CRP and TNFa in frail subjects and hypothesized that these mediators might accumulate in the joints, inducing localized low-grade injury, cartilage destruction and leading to altered metabolism of the joint’s structural breakdown [24,26–28]. Primary OA is considered to be a degenerative disease; however, in the last decade, there has been increasing evidence that OA is a multifactorial disease and that low-grade chronic synovial inflammation plays an important role in OA. Synovial inflammation is mainly mediated by immune cells, particularly macrophages, which are major players in chronic synovial inflammation, bone redundancy formation, arthralgia, subchondral bone remodeling and cartilage damage, and the degree of macrophage activation also correlates with the severity of OA [29,30]. It secretes pro-inflammatory cytokines such as TNFα, IL1, IL6 and IL10, which exert pro-inflammatory effects [31]. A Korean study found that leukocytosis and thrombocytosis in KOA are often accompanied by frailty syndrome, and there is an increased risk of frailty syndrome associated with leukocytosis in KOA [7]. In addition, sedentary behaviour was also associated with an elevated inflammatory marker profile in older adults, a factor that is associated with sarcopenia and frailty [32–34].

Devyani Misra et al. conducted a study suggesting that osteoarthritis of the knee may increase the risk of frailty, with patients suffering from osteoarthritis of the knee having a prevalence of frailty that was 60% to 2 times higher than that of their counterparts, with the prevalence of frailty increasing further as the arthritic condition worsened [15]. Our study corroborates this in the opposite direction. When the Frailty Index (FI) was treated as a continuous variable, univariate logistic analysis indicated that FI is a risk factor for Knee Osteoarthritis (KOA) with an odds ratio (OR) of 3.54 (3.41, 3.68). After adjusting for all covariates, the multivariate logistic results still showed FI to be a risk factor for KOA, with an OR of 3.63 (3.48, 3.78). However, a non-linear relationship exists between the two. This study employed restricted cubic spline analysis to reveal a nonlinear association between FI and KOA, with an inflection point at 0.81: When FI < 0.81, KOA risk increased steeply with rising FI (OR = 18.62); when FI > 0.81, the risk increase slowed significantly (OR = 2.68). This suggests that a FI of 0.81 serves as a threshold for KOA risk stratification. Early interventions should prioritize middle-aged and elderly individuals with FI values near or below this threshold—such as muscle strength training to improve frailty status and prevent progression to high-risk FI ranges. Concurrently, the slower increase in KOA risk observed at FI > 0.81 may stem from reduced activity capacity in this group, which decreases knee joint injury risk and indirectly lowers KOA incidence. This suggests the need for differentiated management strategies tailored to distinct FI intervals.

Moreover, women are prone to becoming debilitated. This may primarily be due to the rapid decline of estrogen in postmenopausal women, which adversely impacts muscle strength, neuromuscular function, and postural stability, thereby increasing the likelihood of debilitation in older women. Estrogen deficiency-induced osteoporosis is also a risk factor for osteoarthritis, making women more susceptible to developing postmenopausal osteoarthritis [35]. There are significant differences in knee kinematics between males and females, with females having a wider range of axial rotation and weaker quadriceps muscles. Weakness has a greater impact on muscle strength and postural stability in women, and women are more likely to experience joint injuries [36,37]. Additionally, women are more likely to eat foods high in sugar and fat, and an unhealthy diet may lead to dysbiosis of the gut flora, which is also more prone to OA including KOA [38].

Similarly, individuals with lower education levels and those residing in rural areas are at a higher risk of suffering from Knee Osteoarthritis (KOA), potentially reflecting disparities in healthcare access. People in these groups are more likely to be involved in strenuous labor and may have a less nutritious diet, with fewer meat, eggs, and dairy products. This can result in weakened musculoskeletal strength. As frailty increases, the intensity of their labor becomes more likely to contribute to the development of KOA [39]. Differences in educational attainment and regional areas of residence suggest that KOA may have a significant negative impact on daily activities and emotional well-being, and the lack of awareness of the disease itself and the adverse consequences it produces in this population is a hidden threat [40]. Enhancing healthcare and targeted KOA education for at-risk populations can reduce its impact. Health interventions should focus on both frail individuals and seemingly healthy yet at-risk people. This study provides a basis for future research on preventive and therapeutic interventions for different frailty levels. The higher KOA occurrence in people without blood pressure and lipid abnormalities may indicate a lack of awareness of frailty-related risks. Those with pre-existing hypertension and hyperlipidemia might reduce activity or do lighter work when frail, while those with normal lipids and blood pressure may continue their usual lifestyle [41]. The shared risk factors of debility and KOA might have a cumulative effect, increasing the likelihood of developing KOA. This implies the need to raise community awareness about frailty and KOA, and that health interventions should target not just frail individuals, but also those who seem healthy yet remain at risk.

Recent longitudinal evidence has begun to unravel the bidirectional nature of the frailty–KOA relationship. In a 2023 community-based cohort of 4,652 U.S. older adults, frailty at baseline doubled the risk of incident KOA over 4 years, whereas new-onset KOA independently increased the subsequent rate of frailty progression by 30% [23]. Similarly, data from the Osteoarthritis Initiative [42] showed that worsening knee pain or radiographic KOA predicted a steeper annual increase in frailty index, even after adjusting for baseline comorbidities and physical activity. These findings align with our cross-sectional observation of a log-linear rise in KOA prevalence across FI quartiles, but they also underscore that the association is unlikely to be unidirectional. Liu et al. and Wen et al. employed Mendelian randomization to demonstrate a causal relationship between KOA and frailty [43,44]. Meanwhile, Tian et al. revealed a longitudinal bidirectional association between knee osteoarthritis and frailty in middle-aged and older adults, particularly highlighting frailty’s dominant role in knee osteoarthritis progression and the cumulative negative impact of knee osteoarthritis on frailty over time [45]. Integrating these longitudinal results suggests a vicious cycle: frailty-related sarcopenia and systemic inflammation may initiate knee-joint degeneration, while chronic pain and mobility restriction from KOA accelerate the decline in physiologic reserve. This bidirectional cycle may be mediated by inflammatory pathways (IL-6, CRP) and imbalance within the “muscle-joint functional unit.” In clinical practice, it is crucial to emphasize early intervention—both to control KOA symptoms and delay frailty progression, and to improve frailty status through nutritional support and exercise training, thereby breaking the vicious cycle between frailty and osteoarthritis. Future studies should therefore model frailty and KOA as time-varying exposures rather than treating either as a static endpoint.

This study constructed six machine learning models to analyze the association between functional impairment (FI) and knee osteoarthritis (KOA). Through random forest feature selection and 5-fold cross-validation, BMI, FI, age, and educational level were identified as key predictors for KOA, providing quantifiable core indicators for clinical KOA risk screening.

Meanwhile, the random forest achieves this by integrating multiple decision trees and conducting random feature selection. It effectively reduces overfitting while maintaining high prediction accuracy, and it possesses stability, robustness and ease of use. This establishes a foundational framework for subsequent exploration of precise interventions tailored to stratified populations. Calibration curves and decision curves validate the model’s clinical utility, demonstrating stable net benefit across a wide range of probability thresholds. This indicates its potential for translation into a clinical risk scoring tool—such as rapidly assessing knee osteoarthritis (KOA) risk in middle-aged and elderly populations by inputting FI and BMI metrics—to support community screening and referral decisions.

Our study still has some limitations. KOA data relied on patient self-report, potentially biased by memory recall, though CHARLS’ trained researchers minimized this. Objective measures like clinical assessments should be included in future studies. There’s a discrepancy between CHARLS’ cognitive tools and the MMSE, hindering direct inclusion of cognitive function in FI calculations, but our 35-item FI aligns with research on accurate outcome prediction. Future studies should use MMSE-compatible assessments for better frailty evaluation. Our cross-sectional design couldn’t establish causality; longitudinal follow-ups could strengthen temporal and causal analyses between FI and KOA. We couldn’t include variables like physical activity, diet, and muscle strength due to CHARLS data limitations; future studies must include these covariates for thorough analyses. Our dataset lacks prescription pain medications, but the wide range of chronic conditions in our FI somewhat compensates for this. The CHARLS study employs patient self-assessment for knee osteoarthritis (KOA), presenting multiple inherent limitations. First, subjective bias is significant. Older adults exhibit individual cognitive differences in perceiving and describing joint discomfort, which can be influenced by pain tolerance and psychological state. Recall bias may also occur, leading to reporting discrepancies. Second, the absence of an objective diagnostic gold standard—lacking imaging (e.g., X-ray) or clinical examination confirmation—prevents differentiation between symptomatic and asymptomatic KOA, increasing the risk of underdiagnosis or overreporting. Third, the assessment dimension is singular; CHARLS-related questions do not elaborate on indicators such as pain intensity or joint function limitations, making it difficult to quantify disease severity. Furthermore, comorbidities like other osteoarthritis conditions or chronic diseases may confound self-reported outcomes. Variations in symptom perception among elderly populations across different regions also reduce cross-population comparability of data, potentially introducing bias into subsequent association analyses. Additionally, due to database limitations, lifestyle factors such as physical activity, diet, and sleep were not included. These factors may introduce residual confounding in the association between FI and KOA by influencing muscle function, inflammation levels, and other mechanisms, thereby affecting the interpretation of the observed association. This KOA study relies on self-reported pain diagnoses, which inherently carry potential recall bias. Due to data limitations, separate sensitivity analyses were not conducted. However, bias was minimized through the use of the standardized CHARLS questionnaire (with a fixed definition of “periodic pain”) and by training investigators to provide standardized guidance during data collection.

Future research should conduct longitudinal studies to understand the causal link between FI and KOA in older populations, targeting frailty progression stages for key intervention points. Intervention studies on techniques like strength training and lifestyle management could assess their effectiveness in reducing KOA risk. Incorporating genetic indicators, muscle mass assessments, and psychological factors could enhance understanding and enable personalized KOA management.

## Conclusion

In conclusion, we document a robust, non-linear association between frailty index and knee osteoarthritis among Chinese adults aged ≥ 45 years. The random forest model (AUC = 0.90) outperformed five alternative algorithms, underscoring its potential for early risk stratification. Nevertheless, causal inferences remain constrained by the cross-sectional design. Longitudinal and bidirectional studies are urgently warranted to clarify whether frailty-reduction strategies (e.g., resistance exercise, nutritional support) can attenuate KOA incidence or progression, and to determine how incident KOA feeds back into frailty trajectories.

## Supporting information

S1 Fig Feature selection was performed using the Random Forest (RF) algorithm and cross-validation. (PDF)

S2 Fig ROC curves and model evaluation of six ML models in validation set. (A): ROC curves; (B): Calibration Curves; (C): Decision curves analysis.
(PDF)

S1 Table Characteristics of participants in training set and a validation set. (DOCX)

S2 Table The multi-dimensional evaluation results for six different machine learning models in the validation set. (DOCX)

S1 Appendix The specific evaluation criteria, items, and scoring methodology for the frailty index. (DOCX)
