# Supplementary figures and images for: Exploring the association between Frailty Index and Knee osteoarthritis in middle-aged and older Chinese adults: A cross-sectional analysis of data from the China Health and Retirement Longitudinal Study
Source: PLoS One. 2026 Mar 27;21(3):e0343370. doi: 10.1371/journal.pone.0343370 (PMC13028503; doi:10.1371/journal.pone.0343370)

Feature Contribution and AUC Performance (Top 4 Features Highlighted)

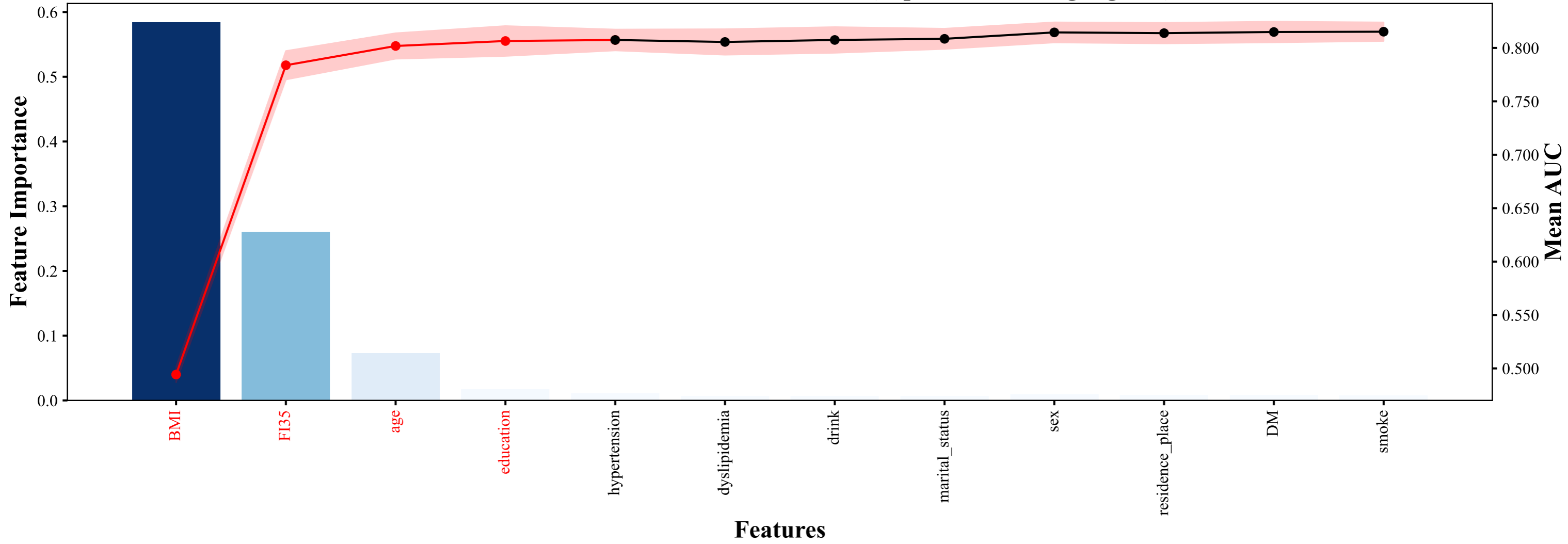

Supplement: S1 Fig — (PDF) [file pone.0343370.s001.pdf]

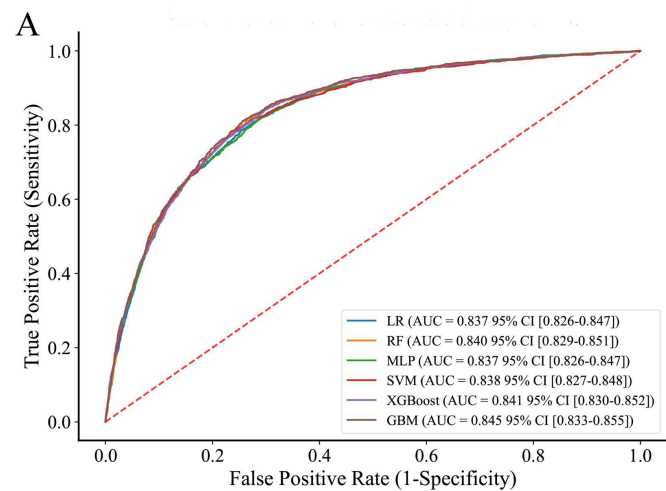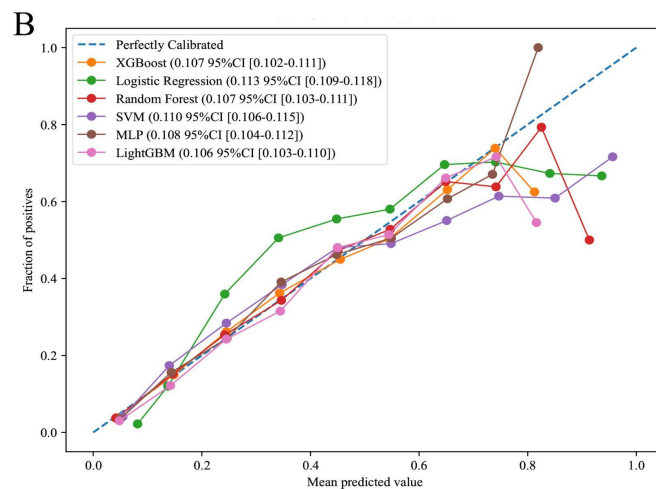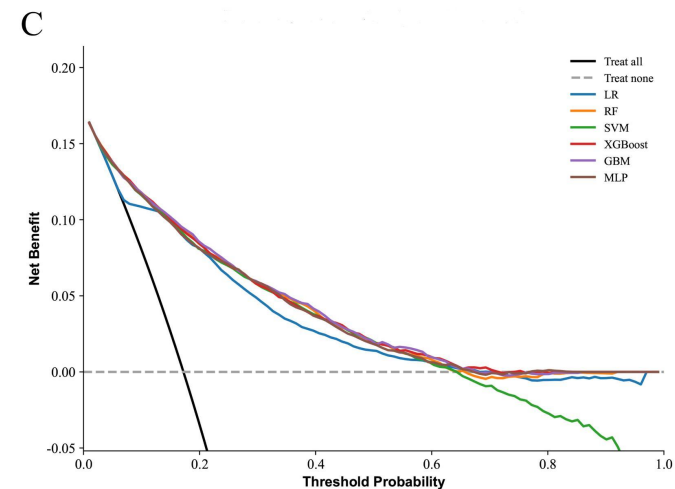

Supplement: S2 Fig — (A): ROC curves; (B): Calibration Curves; (C): Decision curves analysis. (PDF) [file pone.0343370.s002.pdf]
