# Supplementary material for: Exploring the association between Frailty Index and Knee osteoarthritis in middle-aged and older Chinese adults: A cross-sectional analysis of data from the China Health and Retirement Longitudinal Study
Source: PLoS One. 2026 Mar 27;21(3):e0343370. doi: 10.1371/journal.pone.0343370 (PMC13028503; doi:10.1371/journal.pone.0343370)
Supplement: S1 Table — (DOCX) [file pone.0343370.s003.docx]

**S1 Table. Characteristics of participants in training set and a validation set.**

| **Variable** | **Overall** | **Train** | **Validation** | **P-Value** |
| --- | --- | --- | --- | --- |
| N | 29105 | 20373 | 8732 |  |
| Age,year,mean±SD | 60.1 (9.5) | 60.1 (9.5) | 60.2 (9.5) | 1.000 |
| BMI,mean±SD | 23.8 (3.8) | 23.8 (3.8) | 23.8 (3.9) | 1.000 |
| Frailty Index,mean±SD | 0.1 (0.1) | 0.1 (0.1) | 0.1 (0.1) | 1.000 |
| Education,n(%) |  |  |  |  |
| Illiterate | 17951 (61.7) | 12556 (61.6) | 5395 (61.8) | 1.000 |
| Junior high school | 7688 (26.4) | 5371 (26.4) | 2317 (26.5) |  |
| High school or above | 3466 (11.9) | 2446 (12.0) | 1020 (11.7) |  |
