# Supplementary material for: Exploring the association between Frailty Index and Knee osteoarthritis in middle-aged and older Chinese adults: A cross-sectional analysis of data from the China Health and Retirement Longitudinal Study
Source: PLoS One. 2026 Mar 27;21(3):e0343370. doi: 10.1371/journal.pone.0343370 (PMC13028503; doi:10.1371/journal.pone.0343370)
Supplement: S2 Table — (DOCX) [file pone.0343370.s004.docx]

S2 Table. The multi-dimensional evaluation results for six different machine learning models in the validation set.

| Models | Accuracy | Sensitivity | Precision | Specificity | F1 | AUC |
| --- | --- | --- | --- | --- | --- | --- |
| SVM | 0.85 | 0.36 | 0.60 | 0.95 | 0.45 | 0.84 |
| XGBoost | 0.85 | 0.34 | 0.60 | 0.95 | 0.43 | 0.84 |
| LightGBM | 0.85 | 0.34 | 0.60 | 0.95 | 0.44 | 0.84 |
| Logistic Regression | 0.84 | 0.17 | 0.65 | 0.98 | 0.27 | 0.84 |
| Random Forest | 0.85 | 0.34 | 0.60 | 0.95 | 0.43 | 0.84 |
| MLP | 0.85 | 0.35 | 0.59 | 0.95 | 0.44 | 0.84 |
