# Supplementary material for: Exploring the association between Frailty Index and Knee osteoarthritis in middle-aged and older Chinese adults: A cross-sectional analysis of data from the China Health and Retirement Longitudinal Study
Source: PLoS One. 2026 Mar 27;21(3):e0343370. doi: 10.1371/journal.pone.0343370 (PMC13028503; doi:10.1371/journal.pone.0343370)
Supplement: S1 Appendix — (DOCX) [file pone.0343370.s005.docx]

**S1 Appendix. The specific evaluation criteria, items, and scoring methodology for the frailty index.**

| Type of deficit | Item | Variables | Cut-off point |
| --- | --- | --- | --- |
| Activities of daily living | 1 | Bathing | No=0; a little =0.33; need help = 0.67; Yes=1 |
|  | 2 | Dressing | No=0; a little =0.33; need help = 0.67; Yes=1 |
|  | 3 | Use of toilet | No=0; a little =0.33; need help = 0.67; Yes=1 |
|  | 4 | Transferring | No=0; a little =0.33; need help = 0.67; Yes=1 |
|  | 5 | Continence | No=0; a little =0.33; need help = 0.67; Yes=1 |
|  | 6 | Eating | No=0; a little =0.33; need help = 0.67; Yes=1 |
| Instrumental activities of daily living | 7 | Cooking | No=0; a little =0.33; need help = 0.67; Yes=1 |
|  | 8 | Shopping | No=0; a little =0.33; need help = 0.67; Yes=1 |
|  | 9 | Doing household | No=0; a little =0.33; need help = 0.67; Yes=1 |
|  | 10 | Taking medicine | No=0; a little =0.33; need help = 0.67; Yes=1 |
|  | 11 | Managing money | No=0; a little =0.33; need help = 0.67; Yes=1 |
| Physical functional limitations | 12 | Lift a weight of 5 kg | No=0; a little =0.33; need help = 0.67; Yes=1 |
|  | 13 | Walking 1 km | No=0; a little =0.33; need help = 0.67; Yes=1 |
|  | 14 | Walking 100m | No=0; a little =0.33; need help = 0.67; Yes=1 |
|  | 15 | Stooping, kneeling, or crouching | No=0; a little =0.33; need help = 0.67; Yes=1 |
|  | 16 | Able to stand up from sitting | No=0; a little =0.33; need help = 0.67; Yes=1 |
|  | 17 | Able to pick up a coin from a table | No=0; a little =0.33; need help = 0.67; Yes=1 |
|  | 18 | Running or jogging about 1 km | No=0; a little =0.33; need help = 0.67; Yes=1 |
|  | 19 | Reaching or extend arms | No=0; a little =0.33; need help = 0.67; Yes=1 |
|  | 20 | Climbing several flights of stairs without resting | No=0; a little =0.33; need help = 0.67; Yes=1 |
| Chronic disease | 21 | Chronic lung diseases (Chronic bronchitis, emphysema) | Yes=1; no=0 |
|  | 22 | Asthma | Yes=1; no=0 |
|  | 23 | stroke | Yes=1; no=0 |
|  | 24 | CVD | Yes=1; no=0 |
|  | 25 | Gastric or duodenal ulcer | Yes=1; no=1 |
|  | 26 | Kidney disease | Yes=1; no=1 |
|  | 27 | Liver disease | Yes=1; no=1 |
|  | 28 | Memory related disease (Dementia, brain atrophy, and Parkinson's disease) | Yes=1; no=0 |
|  | 29 | Emotional, nervous, or psychiatric problems | Yes=1; no=0 |
| Mental health | 30 | Feel depressed | Always=1; often=0.67; sometimes=0.33; seldom or never=0 |
|  | 31 | Feel fearful | Always=1; often=0.67; sometimes=0.33; seldom or never=0 |
|  | 32 | Feel happy | Always=0; often=0.33; sometimes=0.67; seldom or never=1 |
|  | 33 | Feel everything was an effort | Always=1; often=0.67; sometimes=0.33; seldom or never=0 |
|  | 34 | Feel could not get "going" | Always=1; often=0.67; sometimes=0.33; seldom or never=0 |
| Subjective functioning | 35 | Self-rated health | Very good=0; good=0.25; average=0.5; bad=0.75; very bad=1 |
